# Combination of two long-acting antipsychotics in the treatment of a patient with schizoaffective disorder: a case report

**DOI:** 10.1192/j.eurpsy.2026.11777

**Published:** 2026-07-31

**Authors:** M. Ivandić, B. Uglešić, T. Glavina, M. Galetić, D. Vukorepa, J. Merćep, T. Mastelić

**Affiliations:** Department of Psychiatry, University Hospital of Split, Split, Croatia

## Abstract

**Introduction:**

Long-acting antipsychotics are beneficial for improving adherence in patients with schizophrenia spectrum disorders. The problem of adherence is significant in this population of psychiatric patients. When we add to this the resistance of psychotic disorders to therapy, in a part of patients, it is clear what kind of improvement long-acting antipsychotics have brought. Despite this, two long-acting antipsychotics are rarely combined in the treatment of schizophrenia and similar disorders.

**Objectives:**

Our goal is to present the case of a patient treated for schizoaffective disorder in the Department of Psychiatry, who was treated with two long-acting antipsychotics, which achieved long-term stable remission after a long time.

**Methods:**

Review of medical records.

**Results:**

The patient has been receiving psychiatric treatment since 2011. Since 2014, he has been treated with long-acting olanzapine. Despite this, until 2019, the patient was hospitalized once a year, i.e. 5 times. At the end of 2019, along with long-acting olanzapine, long-acting aripiprazole was introduced into therapy. At the end of 2023, due to the increase in body weight, long-acting olanzapine was replaced by long-acting fluphenazine. For almost 5 years, since the introduction of another long-acting antipsychotic, the patient has had regular follow-ups and is in stable remission, i.e. he has not been hospitalized even once.

**Conclusions:**

As much as one-third of patients suffering from schizophrenia are therapeutically resistant, after changing several forms of therapy. Poor adherence is one of the leading causes of relapse and rehospitalization for patients with schizophrenia. Long-acting injectable antipsychotics can improve adherence and overcome difficulties in offering and maintaining regular clinical follow-ups. However, some studies suggest that long-acting antipsychotics are underused, and in particular, two long-acting antipsychotics are rarely used at the same time. Scientific research so far provides scant data on the simultaneous use of two long-acting antipsychotics. According to the available data, the disadvantages of this practice are pain due to double application, they are usually applied in a hospital, side effects are more difficult to correct, and the medicine cannot be changed quickly. We can say that the mentioned problems are partly present even with the application of one drug. So far, no new or more frequent side effects have been reported when using two long-acting antipsychotics. In our patient, another long-acting antipsychotic proved to be exactly what is needed for long-term stable remission. Given the paucity of data, it is clear that additional research is needed, as well as caution in clinical practice. However, we should not completely reject the simultaneous use of two long-acting antipsychotics.

**Disclosure of Interest:**

None Declared

